# Differential diagnosis of hepatocellular carcinoma and intrahepatic cholangiocarcinoma by ultrasonography combined with multiphase enhanced computed tomography

**DOI:** 10.7150/jca.94550

**Published:** 2024-04-23

**Authors:** HaiYing Tian, Yuling Chen, XiaoHai Li, LiNa Zhao, Sha Li, ChunYan Liao, YeTing Wu, Bei Zhang

**Affiliations:** 1 Clinical Medical College, Guizhou Medical University, Guiyang, Guizhou, 550004, People's Republic of China.; 2 Department of Ultrasound Medicine, Guizhou Provincial People's Hospital, Guiyang, Guizhou, 550002, People's Republic of China.; 3 NHC Key Laboratory of Pulmonary Immune-Related Diseases, Guizhou Provincial People's Hospital, Guiyang, People's Republic of China.; 4 Department of Pathology, Guizhou Provincial People's Hospital, Guiyang, Guizhou, 550002, People's Republic of People's Republic of China.; 5 Department of Radiology, Guizhou Provincial People's Hospital, Guiyang, Guizhou, 550002, People's Republic of People's Republic of China.; 6 Department of Ultrasound Medicine, The Affiliated Hospital of Guizhou Medical University, No. 28 Guiyi Street, Yunyan District, Guiyang, Guizhou, 550004, People's Republic of China.; 7 Department of Infectious Diseases, The Affiliated Hospital of Guizhou Medical University, Guiyang, Guizhou, 550004, People's Republic of China.

**Keywords:** hepatocellular carcinoma, intrahepatic cholangiocarcinoma, ultrasonography, enhanced computed tomography, differential diagnosis

## Abstract

**Purpose:** Hepatocellular carcinoma (HCC) and intrahepatic cholangiocarcinoma (ICC) are primary liver cancers with different therapeutic methods and prognoses. This study aims to investigate the ultrasonography and enhanced computed tomography (CT) features of these cancers and improve the early diagnosis rate.

**Methods:** We retrospectively analyzed the clinical and imaging data of 319 patients diagnosed with HCC and 124 patients diagnosed with ICC, confirmed by pathology.

**Results:** A total of 443 patients were eligible in this study. From the perspective of clinical data, between HCC and ICC patients existed significant differences in age, gender, hepatic background, serum tumor markers of AFP and CA19.9, chronic hepatitis B/C and lymph node infiltration (p<0.05), but not in tumor size, microvascular invasion, serum tumor markers of CEA and CA125 (P>0.05). With respect to ultrasonography features, HCC patients had a higher proportion than ICC patients in splenomegaly (p=0.001), while ICC patients had a higher proportion than HCC patients in absence/not rich vascularity and intrahepatic bile duct dilatation (p<0.05). With respect to CT features, HCC patients were significantly different from ICC patients in the three-phase enhanced CT value mean, enhanced intensity and homogeneity of nodules (P<0.05). A multivariate logistic regression analysis was performed to further clarify the correlation of these indices. However, only age≤60 years (OR=1.861, P=0.045), male (OR=3.850, P<0.001), AFP>7ng/ml (OR=0.119, P<0.001), lymph node infiltration (OR=5.968, P<0.001), intrahepatic bile duct dilatation (OR=2.414, P=0.04), splenomegaly (OR=0.081, P<0.001), rim APHE (OR=3.109, P=0.002), and iso- or hyper enhancement (OR=0.188, P<0.001) were independent risk factors.

**Conclusions:** While there are overlapping ultrasonography and CT features between HCC and ICC, the integration of tumor markers and specific imaging characteristics can be beneficial in distinguishing between the two.

## Introduction

Primary liver cancer (PLC) poses a significant global health burden, ranking as the sixth most prevalent cancer and the fourth leading cause of cancer-related deaths worldwide 1. Among the various types of PLCs, hepatocellular carcinoma (HCC) and intrahepatic cholangiocarcinoma (ICC) are the most common, accounting for approximately 70% and 15% of cases, respectively 2,3. Despite sharing similar risk factors such as viral hepatitis and cirrhosis, the prognosis and long-term outcomes differ significantly between HCC and ICC, with ICC generally having a poorer prognosis 4.

Currently, treatment strategies for HCC, as outlined in guidelines, include surgical resection, transplantation, and percutaneous ablation 5. In contrast, resection is considered the primary treatment approach for ICC 6. Therefore, accurate differentiation between HCC and ICC is crucial for appropriate clinical management and prognostic prediction.

Cross-sectional imaging techniques such as dynamic contrast-enhanced ultrasound (D-CEUS), multiphase enhanced computed tomography (CT), and multiparametric magnetic resonance imaging (MRI) play a vital role in the diagnosis, staging, treatment response evaluation, and surveillance of hepatic carcinoma 7. However, non-invasive differentiation between HCC and ICC remains a significant challenge 8.

Currently, there is limited published research focusing on the differences in ultrasonography and CT features between patients with HCC and ICC. Therefore, this study aims to specifically analyze and compare the ultrasonography and multiphase enhanced CT features of nodules in HCC and ICC patients. The objective is to provide valuable insights for early and accurate clinical diagnosis and to establish standardized treatment approaches.

## Materials and Methods

### Patient selection and study design

The Ethics Committees approved this retrospective research in the Guizhou Provincial People's Hospital (No: 2023-095). Informed written consent of patients was waived. The study involved reviewing clinical medical data of patients diagnosed with pathologically confirmed PLCs between October 2021 and June 2023. The inclusion criteria for this study were as follows: (1) hepatic nodules were pathologically confirmed as HCC or ICC; (2) ultrasonography and multiphase enhanced CT scans were performed within 14 days before the operation; (3) patients did not receive any preoperative anticancer treatments such as radiotherapy or systemic chemotherapy, etc. Patients with poor imaging quality and incomplete clinical information were excluded from the study. In cases where multiple lesions were present, the dominant tumor was selected for analysis. A total of 319 patients with HCC and 124 patients with ICC were included in the study. All data were obtained from the clinical electronic medical record system in accordance with relevant guidelines and approved by our hospital. Patient records were anonymized and deidentified prior to analysis. The pathological results were initially observed and diagnosed by a physician with over 5 years of pathological experience and then reviewed by an expert with over 10 years of experience. Baseline clinical data, including age, gender, cirrhosis status, hepatitis status, and serum tumor marker levels (AFP, CA19.9, CA125, and CEA), were obtained from medical records.

### Hepatic ultrasonography

The Aixplorer Sxc6-1, SonoScape S60, and Esaote MyLab 9 ultrasonic diagnostic instruments were utilized in this study, with a probe frequency of 2-6 MHz. Experienced technicians, who were unaware of the study design, performed hepatic ultrasonography. Two experienced ultrasound physicians independently analyzed the images using a double-blind method. Various ultrasonography features were examined, including the number, location, size, boundary, sharpness, internal echo, and presence of capsular invasion. Additionally, intrahepatic bile duct dilatation and splenomegaly were observed. Following conventional grayscale ultrasonography, color doppler flow imaging (CDFI) was employed to identify the presence or absence of perinodular and intramodular vascular distributions. Based on the echo characteristics of the lesions, the data were classified into three categories: 1) hyperechoic: echo intensity higher than that of normal liver parenchyma; 2) hypoechoic: echo intensity lower than that of normal liver parenchyma; 3) mixed: the presence of a mixture of high and low echoes within a nodule, and even the occurrence of a hypoechoic fluid-filled area. According to the classification of Adler Grades of Blood Flow 9, the images were further categorized as follows: 1) grade-0, no blood flow signal; 2) grade-I, a small amount of blood flow with 1 or 2 dot-like or short rod blood flow signals; 3) grade-II, moderate blood flow with 3 or 4 dot-like or 1 longer blood vessel in the lesion, with a length and diameter approaching or exceeding the radius of the lesion; 3) grade-III, rich blood flow with ≥5 visible dot-like or 2 long blood vessels. In this study, grades 0-I were classified as absence/not vascularity, while grades II-III were classified as rich vascularity.

### Hepatic enhanced CT examination

Preoperative enhanced CT images were acquired using two different scanners: the 320-slice spiral Aquilion ONE CT scanner and the 256-slice GE Revolution CT scanner. The CT parameters used were 120 kVp, 200-400 mAs, and 0.6 mm × 64 section collimation, with a single breath-hold spiral acquisition. Prior to contrast agent administration, unenhanced images were collected. Subsequently, each patient was administered a non-ionic iodinated contrast agent (Iodixanol, 370 mg I/mL, Bracco) via a power injector based on their body weight (2.0 mL/kg, maximum dose of 180 mL), followed by a 20 mL saline flush. Three-phase high-contrast images were acquired immediately after contrast agent administration. The hepatic arterial phase (AP) images were acquired at 25-30 seconds, the portal venous phase (PVP) images were acquired at 60-70 seconds, and the equilibrium phase (EP) images were acquired at 5 minutes 10. All collected images were independently reviewed by a committee of two radiologists with 5 and 15 years of liver imaging experience, who are certified by the board. During the review process, these radiologists remained blind to all patient information.

When analyzing the enhancing features of lesions, several parameters were taken into consideration: (a) attenuation at CT compared with the adjacent liver parenchyma; (b) pattern of enhancement in the arterial, portal/venous, and equilibrium phases; and (c) dynamics of enhancement from the arterial phase to the equilibrium phase. The arterial phase was further divided into three subcategories: (1) arterial phase hyperenhancement (APHE): this refers to the enhancement of the nodule, occupying close to 100% of its volume; (2) partial nodule enhancement: refers to the enhancement of more than 25% of the nodule volume; (3) peripheral hyperenhancement: refers to the enhancement of less than 25% of the nodule volume, resembling a rim-like pattern. For the portal venous and equilibrium phases, the lesions were classified as: (1) hypoenhancement: this indicates lower attenuation compared to the adjacent hepatic parenchyma; (2) iso- or hyperenhancement: this indicates the same or higher attenuation as the adjacent hepatic parenchyma; (3) delayed enhancement on images: This refers to a gradual increase in attenuation over time, surpassing the attenuation of the hepatic parenchyma. These parameters were used to characterize the enhancing features of lesions and provide valuable information for their identification and analysis.

### Statistical analysis

The statistical analysis was conducted using SPSS statistical software, version 25.0 (IBM, Armonk, NY, USA). To determine the significance of differences in continuous variables, the Wilcoxon rank-sum test was employed for skewed data, while Student's t-test was used for normally distributed data. For categorical variables, the Chi-square test or Fisher's exact test was used. Logistic regression analysis was performed to predict the correlation between ultrasonography and CT characteristics, serum tumor markers, and the presence of HCC or ICC. Receiver operating characteristic (ROC) curve analysis was used to assess the diagnostic capability of imaging features and tumor markers in differentiating between HCC and ICC. All p-values were two-sided, and a value of less than 0.05 was considered statistically significant.

## Results

After the strict inclusion, a total of 443 nodules (319 HCC and 124 ICC) in 443 patients were included. In the demographic and clinical data (**Table 1**), significant differences were found between patients with HCC and ICC in age [(54.40±11.52) years old vs. (60.69±10.78) years old], gender ratio (male/female: 263/56 vs. 73/51), lymph node infiltration (7.2% vs. 16.9%), and chronic hepatitis B/C (52.0% vs. 35.5%), p < 0.05. Cirrhosis was more common in the HCC group (48.9% vs 28.2% in ICC lesions), whereas the serum tumor marker of CA19-9 was higher in the ICC patients (35.5% vs 21.6% in HCC lesions, p=0.003), while AFP was significantly lower in ICC than in HCC (P=0.005). The difference in microvascular invasion (45.1% vs. 50.8%) was not statistically significant (X^2^=1.151, p=0.283). The mean sizes of HCC and ICC were 5.89±3.93 cm and 5.61±2.77 cm, respectively (P=0.909).

As shown in **Table 2**, in terms of ultrasonography characteristics, HCC was higher than ICC in the absence/not rich vascularity (91.8% vs. 84.7%) and splenomegaly (41.4% vs. 25.0%), with a statistically significant difference (P<0.05). Compared with HCC, intrahepatic bile duct dilation is more commonly observed in ICC, and this difference was statistically significant (X^2^=18.428, p<0.001). Moreover, there were no significant differences between HCC and ICC groups in terms of gray scale echogenicity, boundary, morphology, or capsular invasion on conventional ultrasound (P>0.05).

**Figure 1:**
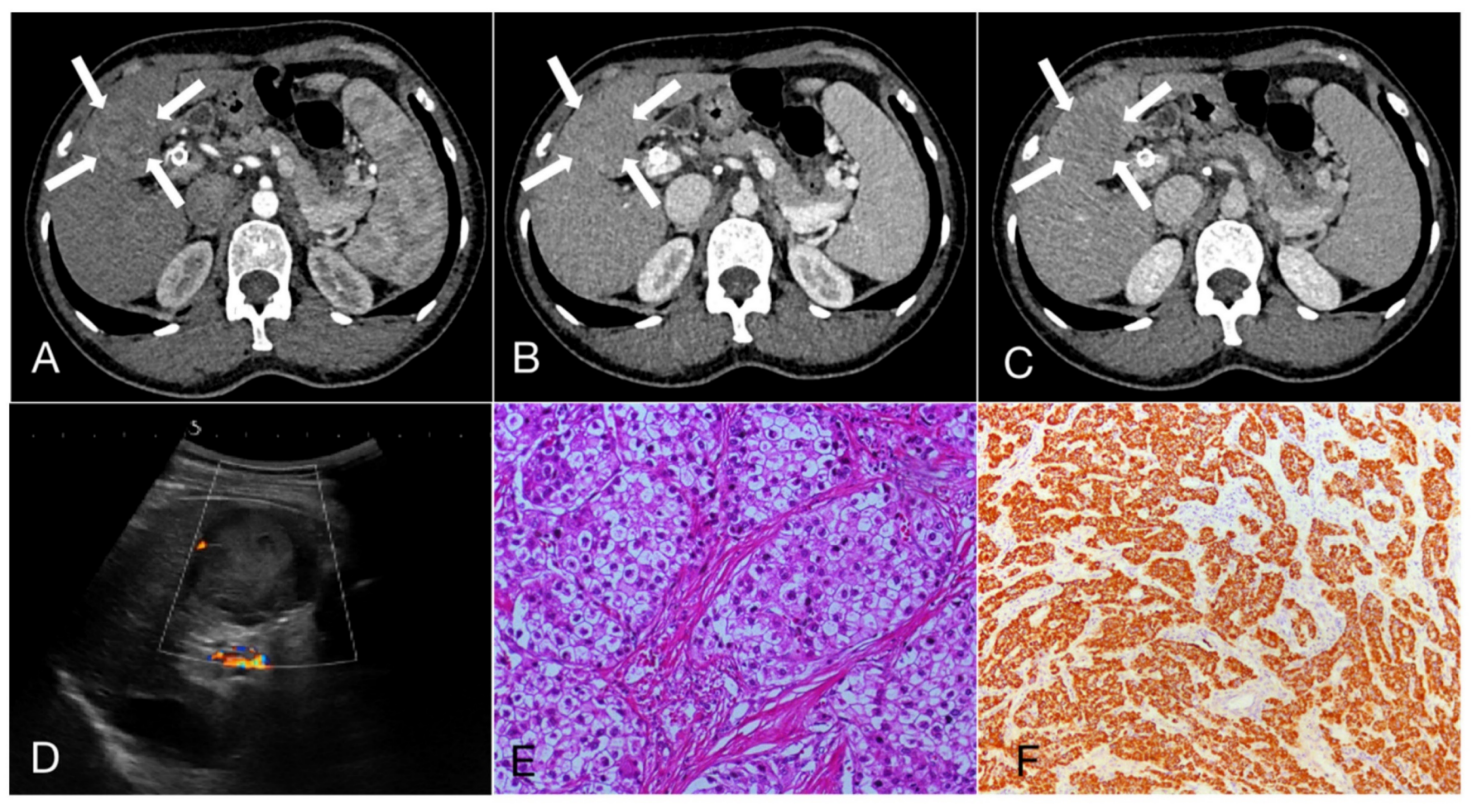
A 55-year-old female patient with hepatocellular carcinoma (3.7cm×3.3cm), accompanied by liver cirrhosis and splenomegaly. **A, B, C.** In multiphase enhanced computed tomography, the nodules exhibit overall hyperenhancement during the arterial phase, with indistinct regression during the portal and equilibrium phases, showing Iso- or hyper enhancement. **D.** Ultrasound demonstrated a mixed echo nodule in the segment 4 of the liver with Grade-I of blood flow; **E.** Hematoxylin and eosin staining, ×200; **F.** The immunohistochemical marker Hepatocyte, ×100.

**Figure 2:**
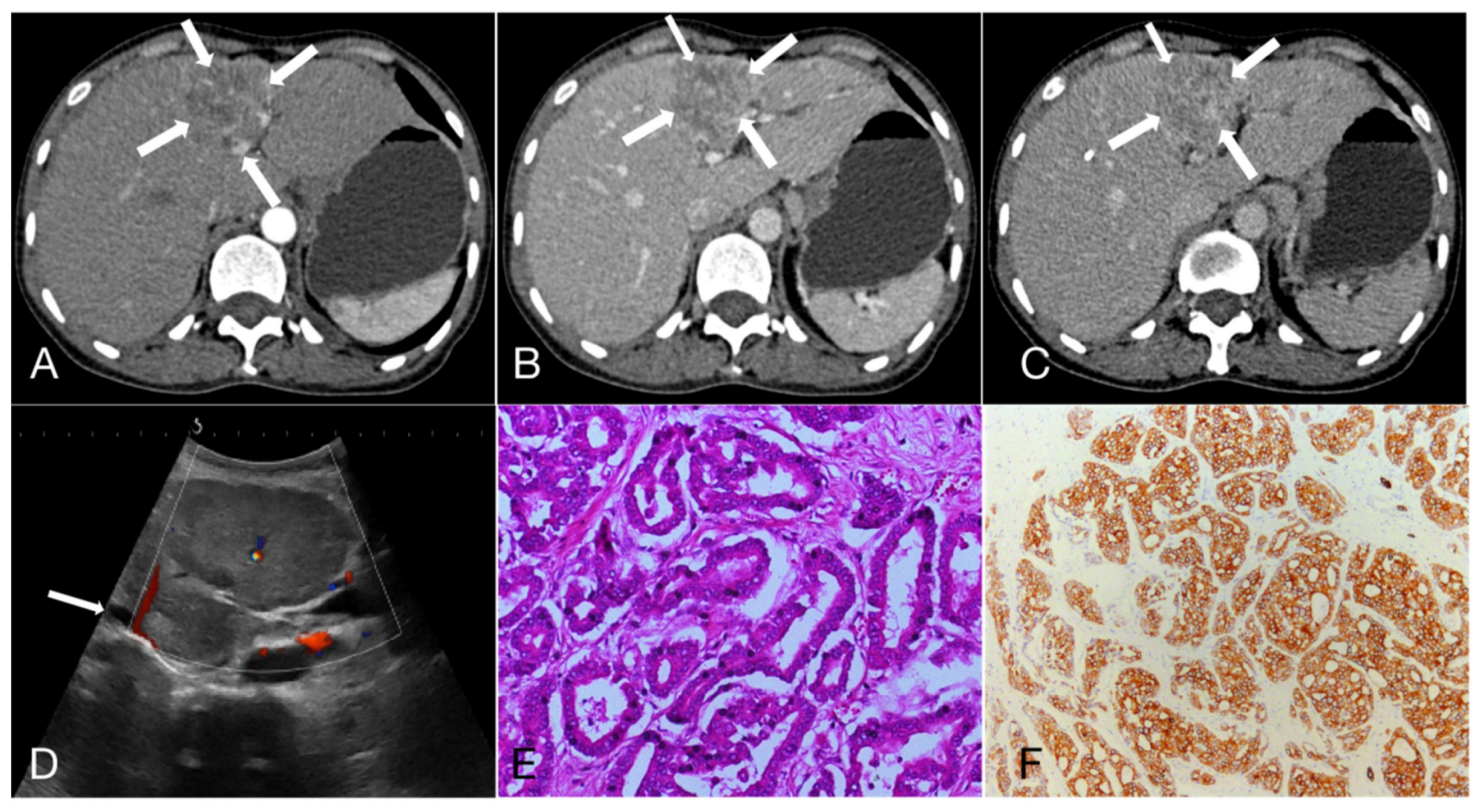
A 47-year-old female patient with Intrahepatic cholangiocarcinoma (6.9cm×5.6cm). **A, B, C.** Rim arterial phase hyperenhaancement (APEH) (arrow) in the arterial phase, followed by early portal venous phase washout; **D.** Ultrasound demonstrated a moderately echogenic nodule in the segment 4 of the liver, and the dilated intrahepatic bile ducts can be observed (arrow); **E.** Hematoxylin and eosin staining, ×200; **F.** The immunohistochemical marker CK7, ×100.

As shown in **Table 3**, 47.3%, 22.3% and 30.4% of HCC showed APHE, partial APHE, and rim APHE in the arterial phase, while the percentages of ICC with these imaging features were 30.6%, 37.1%, and 32.3%, respectively (X^2^ = 13.374, P=0.001). Hypo-enhancement, delayed enhancement and iso- or hyper-enhancement in the portal and equilibrium phases of CT were observed in 37.6%, 19.5% and 42.9% of HCC, and 58.1%, 25.0% and 16.9% of ICC, respectively. Statistical significance of image features in the portal and equilibrium phases was observed between the two groups (X^2^=11.222, P=0.001). In brief, peripheral rim-like APHE was more commonly observed in ICC, while early washout in the portal venous phase and equilibrium phase was also more prominent (**Figure 1, 2**).

**Figure 3:**
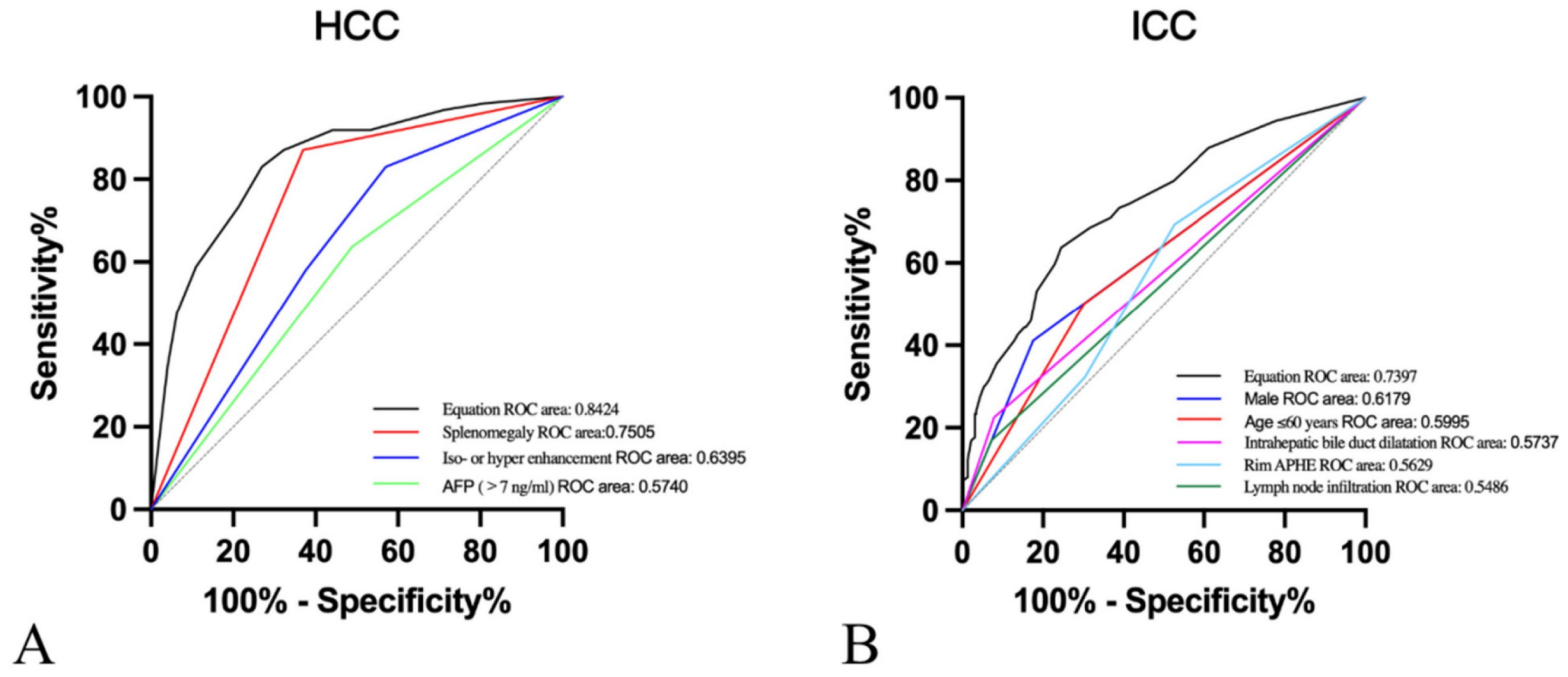
Receiver operating characteristic (ROC) curves of **A.** Splenomegaly (area under the ROC curve [AUROC]=0.7505), iso- or hyper enhancement (AUROC=0.6395), AFP>7ng/ml (AUROC=0.5740), and equation (AUROC=0.8424) for the prediction of HCC. **B.** Male (AUROC=0.6179), Age≤60years (AUROC=0.5995), Intrahepatic bile duct dilatation (AUROC=0.5737), rim APHE (AUROC=0.5629), lymph node infiltration (AUROC=0.5486), and equation (AUROC=0.7397) for the prediction of ICC.

Moreover, after the twelve variables covering age ≤ 60 years, male, cirrhosis, chronic hepatitis B/C, elevated AFP levels, elevated CA19.9 levels, lymph node infiltration, absence/not rich vascularity, intrahepatic bile duct dilatation, splenomegaly, rim APHE and iso- or hyper-enhancement were included for multivariate logistic regression analysis. In our research, predictive risk factors, including age≤60 years (OR=1.861, P=0.045), male (OR=3.850, P<0.001), AFP>7ng/ml (OR=0.119, P<0.001), lymph node infiltration (OR=5.968, P<0.001), intrahepatic bile duct dilatation (OR=2.414, P=0.04), splenomegaly (OR=0.081, P<0.001), rim APHE (OR=3.109, P=0.002), and iso- or hyper enhancement (OR=0.188, P<0.001), were as shown in **Table 4**. Then, ROC curves were plotted, and the diagnostic value of the risk factors was discriminative with areas under the ROC curves (**Figure 3**). The equation demonstrated a discriminative diagnostic value, as indicated by the area under the ROC curve, which was 0.8424 (95%CI: 0.8015-0.832) for the prediction of HCC and 0.7397 (95%CI: 0.6874-0.7920) for the prediction of ICC.

## Discussion

HCC is the most prevalent type among these two PLCs, while the incidence of ICC has been consistently increasing in recent years, particularly among Asian populations compared to Europe and America. The complex disease characteristics and prognosis pose significant challenges to the treatment of liver cancer. Therefore, enhancing non-invasive diagnostic criteria holds immense clinical significance in the accurate diagnosis and treatment of liver cancer 11.

In the early stages, PLCs often present no specific clinical symptoms and may only manifest as mild changes in liver function. It is frequently detected incidentally as a solitary liver mass during imaging examinations. As the disease progresses, patients may experience various symptoms, including abdominal discomfort, abdominal pain, fatigue, nausea, fever, and the presence of an upper abdominal mass. Jaundice, although less common, can also occur 12,13. Serum biomarker examination is commonly used for the diagnosis of liver cancer. CA19.9 and CEA are frequently utilized serum biomarkers for diagnosing ICC. Although their specificity is not ideal, they still provide value in aiding diagnosis and treatment. The results of this study demonstrated that increased levels of CA19.9 are more commonly associated with ICC, whereas elevated AFP levels suggest HCC in the appropriate clinical setting. Both biomarkers are independent diagnostic factors. Furthermore, research has reported that in patients with a history of primary sclerosing cholangitis (PSC), a CA19.9 level >100 U/mL shows sensitivities and specificities of 75% and 80%, respectively, for diagnosing ICC. However, in patients without a history of PSC, the sensitivity is only 53% 14,15. A further retrospective study demonstrated that the postoperative dynamic monitoring of CA19.9 holds significant value in assessing residual tumor or recurrence and predicting patient prognosis 16. A recent study demonstrated that elevated serum CEA levels (CEA cutoff value, 5 IU/ml) as well as elevated serum CA 19.9 levels (CA 19.9 cutoff value, 37 IU/ml) were found in patients who had locally advanced (p < 0.001) or metastatic (p < 0.001) ICC compared with those who had earlier stage, liver-confined disease 17. Distinguishing between HCC and ICC poses a challenge. In patients with chronic liver diseases, an elevated AFP level, in particular, suggests a greater likelihood of HCC than ICC 18,19.

Ultrasound examination is a widely utilized, non-invasive technique in clinical practice for liver imaging. The grayscale ultrasound examination does not consistently show uniform imaging manifestations of liver cancer. If a liver mass is accompanied by peripheral bile duct dilation, internal calcification, or a solid mass within the dilated bile duct, the presence of ICC should be considered. In the presence of liver cirrhosis or splenomegaly, the possibility of HCC should be considered. Nonetheless, these results frequently lack clinical specificity 20,21. ICC is commonly characterized by hypovascularity during color Doppler ultrasound examination. Real-time contrast-enhanced ultrasound or multiphase CT enhancement examination enables continuous monitoring of the lesion's blood supply status, facilitating qualitative diagnosis of liver lesions. Contrast-enhanced examination reveals typical imaging findings of ICC, such as significant enhancement of the lesion margin in the arterial phase and subsequent rapid clearance. In HCC, enhancement scans commonly exhibit substantial homogeneous enhancement in the arterial and portal venous phases, with mild attenuation in the delayed phase, approaching or slightly lower than the surrounding liver parenchyma 22,23. The study findings additionally confirmed the occurrence of intrahepatic bile duct dilation in ICC, which presents as hypovascularity in ultrasound. Moreover, the nodules demonstrate significant peripheral enhancement during the arterial phase, which can serve as an independent predictive factor. Conversely, HCC frequently presents with splenomegaly, and its nodules exhibit uniform enhancement during the arterial phase, followed by delay or isoenhancement in the portal venous phase.

Iavarone *et al.* 24 conducted a retrospective analysis from two medical centers, providing a comprehensive depiction of the vascular dynamic pattern of ICC using perfusion CT. Of particular interest is the observation that 50% of the ICC lesions exhibited peripheral hyperdensity in the arterial phase, especially in nodules larger than 3 cm. The remaining nodules displayed various appearances, with 16% showing global hyperdensity, 5% partial hyperdensity, 11% isodensity, and 18% hypodensity. Approximately 42% of the lesions demonstrated a progressive increase in contrast enhancement across different phases. Consequently, 45% of the lesions were globally hyperdense during the delayed phase, with 16% showing peripheral hyperdensity and 16% isodensity. Surprisingly, 23% of the lesions even exhibited hypodensity. It is worth noting that none of the lesions exhibited the typical radiological pattern associated with HCC, characterized by global hyperenhancement during the arterial phase and hypoenhancement in the delayed phase according to CT scans. The differentiation between ICC and HCC relies on the contrast enhancement pattern displayed by the lesion. ICC typically presents a portal or delayed-phase enhancement pattern, as its blood supply comes from portal vein branches. On the other hand, HCC receives its blood supply from hepatic arteries, resulting in an arterial-phase enhancement pattern 25. The key distinguishing feature of ICC is the initial rim enhancement or peripheral enhancement observed in the arterial phase, followed by centripetal enhancement in the delayed phase 20. This crucial phase, occurring approximately 3-5 minutes after contrast agent injection, plays a vital role in the diagnosis of ICC, as it is characterized by a prominent central enhancement attributed to the presence of abundant fibrotic tissue 26.

In recent years, there has been significant progress in the application of radiomics in cancer research, which has led to improved non-invasive characterization of lesions 27. A number of studies have focused on utilizing radiomics to analyze liver tissue and tumors in imaging studies 28,29. Specifically, researchers have extensively explored the potential of radiomics features in discriminating between HCC and benign liver lesions. Radiomics involves extracting high-dimensional quantitative features from medical imaging data. This computational technique has the ability to provide accurate descriptions of tumor subtypes, intra- and intertumoral heterogeneity, and clinical outcomes, surpassing the capabilities of conventional imaging. Additionally, it may offer non-invasive surrogate biomarkers 27,30,31. When combined with machine learning, radiomics can serve as a reliable tool for predicting cancer outcomes beyond visual assessment 32. In future research, there will be a continued focus on radiomics, with the aim of improving early diagnosis capabilities in liver cancer and maximizing patient benefits.

Our study has several limitations that should be acknowledged. Firstly, being a retrospective study, there are inherent limitations in terms of limited sample size and potential selection bias. Furthermore, the evaluation of ultrasonography and CT features may be subjective, introducing a degree of variability. Secondly, our study focused exclusively on ICC and HCC, neglecting other hepatic malignancies such as combined hepatocellular-cholangiocarcinoma and metastatic tumors. Future studies should aim to include a broader range of hepatic malignancies to provide a more comprehensive understanding of the topic. Additionally, it is crucial to validate the findings of our study through further research. Furthermore, to enhance the quality of future studies, it is recommended to increase sample sizes in later stages and explore the potential of incorporating new technologies such as contrast-enhanced ultrasonography, sonographic elasticity, and radiomics.

## Conclusion

This study aimed to investigate the clinical relevance of ultrasonography and multiphase enhanced CT features in the differentiation of HCC and ICC. Each modality presents unique characteristics in terms of ultrasonography and CT findings. The integration of both techniques enhances the early detection rate of these two diseases and provides valuable insights for clinical diagnosis and treatment. Accurate preoperative diagnosis plays a vital role in formulating optimal treatment strategies and improving patient prognosis.

## Figures and Tables

**Table 1:** Comparison of clinical features between patients with HCC and ICC.

Clinical features	HCC	ICC	P value
	(n=319, %)	(n=124, %)	
Age, mean±SD (years)	54.40±11.52	60.69±10.78	<0.001
≤60	223 (69.9)	62 (50.0)	
>60	96 (30.1)	62 (50.0)	
Gender			<0.001
Male	263 (82.4)	73 (58.9)	
Female	56 (17.6)	51 (41.1)	
Tumor size, mean±SD (cm)	5.89±3.93	5.61±2.77	0.909
≤5	182 (57.1)	70 (56.5)	
>5	137 (42.9)	54 (43.5)	
Hepatic background			<0.001
Normal	163 (51.1)	89 (71.8)	
Cirrhosis	156 (48.9)	35 (28.2)	
Chronic hepatitis B/C			0.002
Positive	166 (52.0)	44 (35.5)	
Negative	153 (48.0)	80 (64.5)	
Serum tumor markers			
AFP (>7 ng/ml)	163 (51.1)	45 (36.3)	0.005
CEA (>5 ng/ml)	22 (6.9)	14 (11.3)	0.129
CA19.9 (>39 U/ml)	69 (21.6)	44 (35.5)	0.003
CA125 (>35 U/ml)	62 (19.4)	32 (25.8)	0.141
Microvascular invasion			0.283
Yes	144 (45.1)	63 (50.8)	
No	175 (54.9)	61 (49.2)	
Lymph node infiltration			0.002
Yes	23 (7.2)	21 (16.9)	
No	296 (92.8)	103 (83.1)	

**Table 2:** Comparison of ultrasonography characteristics of HCC and ICC.

Characteristic	HCC	ICC	P value
	(n=319, %)	(n=124, %)	
Gray scale echogenicity			0.233
Hyperechoic	81 (25.4)	24 (19.4)	
Hypoechoic	133 (41.7)	62 (50)	
Mixed	105 (32.9)	38 (30.6)	
Boundary			0.247
Clear	81 (25.4)	25 (20.2)	
Unclear	238 (74.6)	99 (79.8)	
Morphology			0.395
Regular	80 (25.1)	36 (29.0)	
Irregular	239 (74.9)	88 (71.0)	
Vascularity			0.025
Absence/not rich vascularity	293 (91.8)	105 (84.7)	
Rich vascularity	26 (8.2)	19 (15.3)	
Intrahepatic bile duct dilatation			<0.001
Yes	25 (7.8)	28 (22.6)	
No	294 (92.2)	96 (77.4)	
Capsular invasion			0.368
Yes	191 (59.9)	80 (64.5)	
No	128 (40.1)	44 (35.5)	
Splenomegaly			0.001
Yes	132 (41.4)	31 (25.0)	
No	187 (58.6)	93 (75.0)	

**Table 3:** Comparison of CT features of HCC and ICC.

CT features	HCC	ICC	Statistics	P value
	(n=319, %)	(n=124, %)		
Arterial phase CT value (HU) mean	84.03±22.91	64.79±14.58	t=8.691	<0.001
Portal venous phase CT value (HU) mean	96.48±14.55	87.44±27.22	t=4.504	<0.001
Equilibrium phase CT value (HU) mean	81.29±13.23	74.42±21.26	t=4.087	<0.001
Arterial Phase^a)^			X2=13.374	0.001
-APHE	151 (47.3)	38 (30.6)		
-Partial APHE	71 (22.3)	46 (37.1)		
-Rim APHE	97 (30.4)	40 (32.3)		
Portal and equilibrium phases^b)^			X2=11.222	0.001
Hypo-enhancement	120 (37.6)	72 (58.1)		
Delayed enhancement	62 (19.5)	31 (25.0)		
Iso- or hyper enhancement	137 (42.9)	21 (16.9)		

APHE: Arterial phase hyperenhancement; ^a)^Comparison between APHE and Rim APHE. ^b)^Comparison between Hypo-enhancement and Iso- or hyper enhancement.

**Table 4:** Multivariate Logistic regression analysis of HCC and ICC.

	OR	95% CI		P value
		Low Upper		
Age≤60 years	1.861	1.014	3.413	0.045
Male	3.850	1.995	7.430	<0.001
Cirrhosis	2.506	0.939	6.687	0.066
Chronic hepatitis B/C	1.107	0.554	2.211	0.774
AFP (>7 ng/ml)	0.119	0.048	0.298	<0.001
CA19.9 (>39 U/ml)	1.726	0.899	3.314	0.101
Lymph node infiltration	5.968	2.232	15.957	<0.001
Absence/not rich vascularity	2.294	0.915	5.755	0.077
Intrahepatic bile duct dilatation	2.414	1.039	5.606	0.040
Splenomegaly	0.081	0.040	0.164	<0.001
Rim APHE	3.109	1.517	6.374	0.002
Iso- or hyper enhancement	0.188	0.091	0.389	<0.001
